# Large-Scale Samples Based Rapid Detection of Ciprofloxacin Resistance in *Klebsiella pneumoniae* Using Machine Learning Methods

**DOI:** 10.3389/fmicb.2022.827451

**Published:** 2022-03-08

**Authors:** Chunxuan Wang, Zhuo Wang, Hsin-Yao Wang, Chia-Ru Chung, Jorng-Tzong Horng, Jang-Jih Lu, Tzong-Yi Lee

**Affiliations:** ^1^Warshel Institute for Computational Biology, The Chinese University of Hong Kong, Shenzhen, China; ^2^School of Data Science, The Chinese University of Hong Kong, Shenzhen, China; ^3^School of Life Sciences, University of Science and Technology of China, Hefei, China; ^4^Department of Laboratory Medicine, Chang Gung Memorial Hospital at Linkou, Taoyuan, Taiwan; ^5^Ph.D. Program in Biomedical Engineering, Chang Gung University, Taoyuan City, Taiwan; ^6^Department of Computer Science and Information Engineering, National Central University, Taoyuan, Taiwan; ^7^Department of Bioinformatics and Medical Engineering, Asia University, Taichung City, Taiwan; ^8^Department of Medical Biotechnology and Laboratory Science, Chang Gung University, Taoyuan, Taiwan; ^9^Department of Medicine, College of Medicine, Chang Gung University, Taoyuan, Taiwan

**Keywords:** antibiotic susceptibility test, MALDI-TOF MS, machine learning, *Klebsiella pneumonia*, ciprofloxacin resistance

## Abstract

*Klebsiella pneumoniae* is one of the most common causes of hospital- and community-acquired pneumoniae. Resistance to the extensively used quinolone antibiotic, such as ciprofloxacin, has increased in *Klebsiella pneumoniae*, which leads to the increase in the risk of initial antibiotic selection for *Klebsiella pneumoniae* treatment. Rapid and precise identification of ciprofloxacin-resistant *Klebsiella pneumoniae* (CIRKP) is essential for clinical therapy. Nowadays, matrix-assisted laser desorption ionization time-of-flight mass spectrometry (MALDI-TOF MS) is another approach to discover antibiotic-resistant bacteria due to its shorter inspection time and lower cost than other current methods. Machine learning methods are introduced to assist in discovering significant biomarkers from MALDI-TOF MS data and construct prediction models for rapid antibiotic resistance identification. This study examined 16,997 samples taken from June 2013 to February 2018 as part of a longitudinal investigation done by Change Gung Memorial Hospitals (CGMH) at the Linkou branch. We applied traditional statistical approaches to identify significant biomarkers, and then a comparison was made between high-importance features in machine learning models and statistically selected features. Large-scale data guaranteed the statistical power of selected biomarkers. Besides, clustering analysis analyzed suspicious sub-strains to provide potential information about their influences on antibiotic resistance identification performance. For modeling, to simulate the real antibiotic resistance predicting challenges, we included basic information about patients and the types of specimen carriers into the model construction process and separated the training and testing sets by time. Final performance reached an area under the receiver operating characteristic curve (AUC) of 0.89 for support vector machine (SVM) and extreme gradient boosting (XGB) models. Also, logistic regression and random forest models both achieved AUC around 0.85. In conclusion, models provide sensitive forecasts of CIRKP, which may aid in early antibiotic selection against *Klebsiella pneumoniae*. The suspicious sub-strains could affect the model performance. Further works could keep on searching for methods to improve both the model accuracy and stability.

## Background and Introduction

*Klebsiella pneumoniae* (*K. pneumoniae*) is one of the most common hospital- and community-acquired bacterial infections (Ashurst and Dawson, 2021). Extensively used quinolone antibiotics, which include ciprofloxacin, play a significant role in *K. pneumoniae* treatment. Increases in the proportion of ciprofloxacin-resistant *K. pneumoniae* (CIRKP) and the long inspection time of traditional antimicrobial susceptibility testing (AST) could lead to incorrect initial antibiotic treatment that will squander the essential treatment time of patients (Burckhardt and Zimmermann, 2018). Methods for rapid and precise identification of CIRKP are critical for clinical *K. pneumoniae* infection treatment. With the assistance of matrix-assisted laser desorption ionization time-of-flight mass spectrometry (MALDI-TOF MS) technology, inspection time of AST, and strain typing of infectious bacteria could decrease to less than 2 h after cell culture (van Belkum et al., 2017; Florio et al., 2018). Introducing machine learning methods to antibiotic resistance identification could assist in further improving the inspection speed and lowering the cost (Weis et al., 2020) and discovering potential antibiotic markers from MALDI-TOF MS data. In this study, prediction models for CIRKP in the Taiwan area are constructed based on large-scale mass spectrum data and non-spectrometric information of patients.

Although *K. pneumoniae* is normally harmless and can be found in the human intestine, it can cause serious infections in other parts of the body, such as pneumoniae, urinary tract infection, sepsis, etc. Especially, multi-drug resistant and carbapenem-resistant *K. pneumoniae* (CRKP) has become a great threat to public health, whose overall 30-days mortality rate has been reported to be greater than 40% due to the limited antibiotic options after infection (Tumbarello et al., 2012). Thus, most recent studies related to *K. pneumoniae* focus on creating new identification methods and finding potential resistant biomarkers. However, as a broad-spectrum quinolone antibiotic, ciprofloxacin is one of the widely used antibiotics for the treatments of infection caused by *K. pneumoniae* in clinical therapy. The increasing reported CIRKP (Sanchez et al., 2013; Zhou et al., 2016) could also be a severe problem under clinical circumstances. Identification models for different bacterial species and antibiotics are important to validate the feasibility of the generalization of machine learning-based antibiotic resistance detection using MALDI-TOF MS data.

Clinical prescriptions usually heavily depend on the AST result to guide the initial antibiotic selection and avoid inefficient treatment. Traditional AST procedure usually requires 24 h for plate culture and an additional 24 h for the antimicrobial susceptibility testing (Kumar et al., 2009). The delay of efficient antibiotic treatment will increase the mortality rate, and especially if the patient is seriously infected. In recent years, the polymerase chain reaction (PCR) method has been applied to rapidly detect genes of *K. pneumoniae* related to quinolone resistance, such as mutations on type II and type IV DNA topoisomerase genes (Ruiz, 2003; Nordmann and Poirel, 2005). Besides, abnormal expression of outer cytomembrane efflux pump and plasmid-mediated resistance genes are also proved to be quinolone resistance mechanisms and can be detected by genomic and proteomic methods (Martínez-Martínez et al., 1998). However, restriction to the current gene library, high labor intensity, and excessive cost of the tests are still practical problems for generalizing genome tests. Nowadays, utilizing the MALDI-TOF MS could significantly shorten the testing time and lower the inspection cost. Achievements have been made in both identifying antibiotic-resistant bacteria from laboratory plate cultures and directly from the specimens of patients (Clark et al., 2013; Patel, 2015; Singhal et al., 2015; Angeletti, 2016; Arca-Suárez et al., 2017; Sandalakis et al., 2017; Tré-Hardy et al., 2017). In 2016, spectrum peak at 11.109 m/z was confirmed related to plasmid-mediated CRKP with gene (Gaibani et al., 2016). Besides, polypeptide at 3,043 m/z is proved to be a fragment of PBP2a, which participants in the methicillin resistance process of Staphylococcus aureus (MRSA) (Sogawa et al., 2017). Those study results demonstrate that MALDI-TOF MS can find specific mass peaks with potential biological meanings. It may also detect antibiotic resistance profiles of large protein-involved mechanisms in a low mass-to-charge ratio range. Thus, antibiotic-resistant biomarkers obtained from MALDI-TOF MS data may not only serve as evidence for bacterial type identification, but we may also even be able to find resistant bacterial strains with unrevealed mechanisms.

Predicting models constructed by machine learning methods has achieved high accuracy in identifying antibiotic bacterial strains. Taking the identification of MRSA as an example, the support vector machine (SVM) models show identification accuracy of around 90% (Sogawa et al., 2017; Tang et al., 2019), and the model in the study of Liu et al. (2021) shows the area under the receiver operating characteristic (ROC) curve (AUC) of 0.89 for SVM model and 0.87 for random forest model (RF). Especially, according to Wang et al. (2021), a logistic regression (LR) model is trained based on over 20,000 samples and independently validated by another data set with more than 5,000 samples, and finally achieves a predicting AUC of 0.85. Large samples used in their study and external validation highly improve the reliability of the machine learning model. However, most previous studies apply statistical analysis and construct models only based on a small number of samples (usually the total samples are no more than 1,000), which could significantly limit the statistical power of the analysis and the reliability of models.

This study performed an antibiotic resistance analysis and modeling based on 16,697 samples collected from a longitudinal study from June 2013 to February 2018 and AST results were taken as references. Raw spectra data were first preprocessed by peak smoothing and baseline correction. After that, peaks with signal-to-noise ratio of 2 were selected for further analysis. Since *K. pneumoniae* may cause serious infections in various body parts, this study analyzed six common *K. pneumoniae* specimen carriers, which include blood samples (B), body fluid samples (F), wound samples (W), respiratory samples (R), urinary samples (U), and other types (O). Besides, to simulate the real clinical antibiotic resistance predicting problems, patients’ basic information, including gender and age, were also included in model construction, coupling with separating training and testing sets by time. Statistical tests were applied to select significant features for modeling. Final performances achieved predicting AUC of around 0.89 for SVM and extreme gradient descent boosting (XGB) models and AUC 0.85 for LR and RF models. Moreover, this study also included a clustering analysis based on an unsupervised learning method to provide potential information of different *K. pneumoniae* sub-strains in the data set and quantified the influence of suspicious sub-strains on the model performances.

## Materials and Methods

**FIGURE 1:**
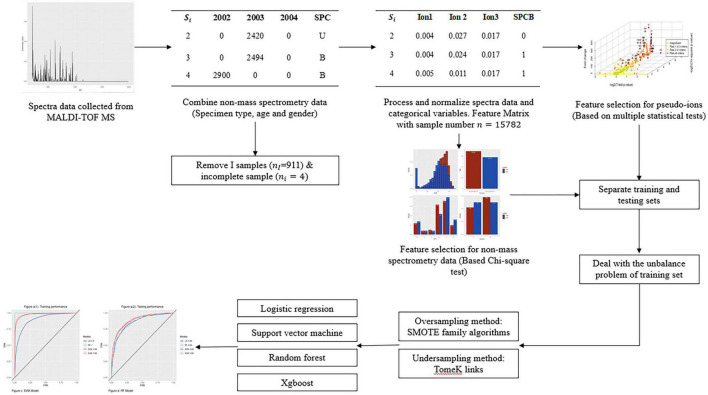
Flow chart of the whole study, including sample collection, data cleaning and processing, feature selection, unbalance problem treatment, and model construction and comparison.

The modeling procedure of this research is shown in the flow chart in Figure 1, which primarily contains five distinct components. The process began with specimen collection and mass spectral data processing, followed by data cleaning for quality control. After that, data processing aimed to unify the spectra’s dimensions and generated dummy variables for categorical variables. Finally, the relevant variables selected in the feature selection step were used to create binary classification models with balancing methods.

### Data Cleaning

The samples used in this study were collected from a longitudinal study on ciprofloxacin that ran from June 2013 to February 2018 done by the Change Gung Memorial Hospitals (CGMH) at the Linkou branch. The longitudinal research included specimens of 16,697 participants in total. To guarantee the data quality, 915 samples labeled as intermediate and 4 incomplete samples were eliminated. In total, 15,782 samples were selected for further analysis, of which 11,354 were ciprofloxacin-susceptible Klebsiella pneumoniae (CISKP) and 4,428 were CIRKP.

### Specimen Preparation and Spectrum Preparation Methods

All the specimen samples were collected as daily routine examinations. Specimens with different carrier types were cultured with the most appropriate methods. Blood specimens were cultured in the trypticase soy broth (Becton Dickinson, MD) and an automated detection system (BD BACTECTM FX; Becton Dickinson) was utilized for detecting positive blood culture results. After that, blood from positive blood culture bottles was inoculated on blood plate (BP) agar for subculture. Fluid specimens and respiratory specimens were inoculated on BP agar, eosin methylene blue (EMB) agar, CNA agar, and chocolate agar. Additionally, some of the fluid specimens were cultured on thioglycolate broth. Specimens obtained from wounds were first rinsed with 1.2 mL of 0.9% saline solution and then inoculated on BP, EMB, CNA, and chocolate agars. For urine specimens, only BP and EMB agars were utilized for the plate culture. All the agars and broths used for specimen cultures were from Becton Dickinson and they were incubated into a 37°C CO2 incubator for 18–24 h. MALDI-TOF MS was performed using selected single colonies. CIRKP specimens were identified from CISKP specimens through the disk diffusion method under the instruction of Clinical and Laboratory Standards Institute guideline M100 (CLSI M100). CISKP samples and CIRKP samples were determined by ATS breakpoints listed in CLSI M100 (Clinical and Laboratory Standards Institute [CLSI], 2018).

The selected colonies were analyzed by MALDI-TOF MS (Microflex LT MALDI-TOF System, Bruker Daltonik GmbH) following the operating instructions created by the manufacturer. First, cultivated colonies were smeared onto the MALDI steel target plate with the addition of formic acid (1 μL, 70%) and then dried at 25°C degrees. Then, a matrix solution (α-cyano-4-hydroxycinnamic acid, 100*mg*/*mL*, 50% acetonitrile with 2.5% trifluoroacetic acid) was added to the spots, and the samples were dried at room temperature. Spectrum data with mass-to-charge ratio (m/z) between 2,000 and 20,000 were then collected using Microflex LT MALDI-TOF analyzer in a linear mode (accelerating voltage, 20 kV; nitrogen laser frequency: 60 Hz; 240 laser shott). The raw spectrum data were first calibrated with an external standard calibrator (Bruker Daltonics Bacterial Test Standard), and then peak smoothing (Savitzky-Golay filter) and baseline correction (Tof-hat filter) were applied. Finally, peaks with signal-to-noise ratio 2 were selected for further analysis.

### Spectrometric Data and Non-spectrometric Data Processing

To unify the dimensions of spectrum data along with alleviating the influence of peak shift, spectral data was grouped into pseudo-ion vectors each with 900 pseudo-ions. Pseudo-ions were calculated by first grouping the mass-to-charge ratio into bins of width 20 m/z, and then selecting the peak with maximum intensity ratio. Using the pseudo-ion peak *k* of the sample *i* as an example:


$$P_{i\,k}=Max_{j}\left(R_{i\,j}\cdot 1_{\left\{\left(2000+20\left(k-1\right)\right)\leq M_{i\,j}<\left(2000+20k\right)\right\}}\right),k\in \left[1,900\right]\cap Z$$

where *M*_*ij*_ and *R*_*ij*_ represent the mass-to-charge ratio and the intensity ratio of the *j^th^* peak of the sample *i*, respectively, 1_{.}_is the indicator function. After that, pseudo-ion vectors were row-bind together to form the spectral data matrix. $\vec{P_{k}}$ indicates the pseudo-ion *k* (the *k^th^* column of the spectral matrix) and the matrix was scaled as


$$\vec{P{}_{k}^{\prime }}=\frac{\vec{P_{k}}-\bar{\vec{P_{k}}}}{\sigma \,\left(\vec{P_{k}}\right)}$$

where $\bar{\vec{P_{k}}}$ and $\sigma \,\left(\vec{P_{k}}\right)$ denote the mean and standard deviation of pseudo-ion *k* in order.

For non-spectrometric features, we grouped the specimen carriers (SPC) into six major types, which included blood sample (B), body fluid sample (F), wound sample (W), respiratory sample (R), urinary sample (U), and other types (O) (Supplementary Table 1). Continuous non-spectrometric feature age was transformed into an ordinal feature with six age groups for the convenience of statistical analysis (Supplementary Table 2). Labels of SPC after grouping and gender were directly used in statistical analysis. Besides, the modeling procedure included dummy variables generated from SPC, gender, and the scaled continuous feature age.

After data processing, the final feature matrix for modeling was formed by dummy variables of SPC, gender, and scaled age, and the pseudo-ion matrix.

### Methods for Statistical Tests and Feature Selection

The Chi-square test was used to determine the impact of non-spectrometric characteristics on the CISKP and CIRKP groups. Non-spectrometric features with *p*-values less than 0.05 were considered as significant and would be included in the modeling.

The significance of pseudo-ions was mainly measured from three perspectives, and all pseudo-ions were tested with their intensity ratio before scaling: (1) the difference in mean values, (2) the difference in standard deviation, and (3) association to CIRKP. In the previous study, the *t*-test could be an option for determining the significance of the mean difference of log-transformed pseudo-ions (Wang et al., 2021). However, *t*-test pre-request normally distributed samples which was not the case in this study (Supplementary Figures 1, 2). Therefore, the non-parametric Wilcoxon rank-sum test was used instead, coupling with the fold change selection on average intensity ratios to capture the information of mean shift of pseudo-ions with few observations. In this study, features with |*log*_2_⁡(*fc*)|≥1 were considered as significant, where *fc* is the fold change value of the average intensity ratio between CIRKP and CISKP groups. As for the test of standard deviation difference, the traditional *F*-test for the equivalence of standard deviation was applied. Finally, the homogenous sample distributions between CIRKP and CISKP were tested by the Kolmogorov-Smirnov test (KS test) to directly measure the association between pseudo-ions and CIRKP. The significant level α=0.05 was used for a decision. Moreover, features were ranked by *p*-values (if the observation time of a feature was insufficient for testing, a *p*-value equal to 1 was set for that feature) of statistical tests, and features were also ranked by the fold change of average intensity ratio in the fold change selection.

The final ranks of pseudo-ions will overall consider all the test results above and take the average ranks of pseudo-ions as the final decision.

### Clustering Method

The clustering method was based on the single-cell clustering approach, which was performed by R 4.1.1 with the assistance of the package “Seurat.” We treat the intensity ratios over mass-to-charge ratio values as the expression level of genes.

### Balancing Methods

For the modeling process in this study, data were first separated into training and testing sets by time, where the training set contained samples from June 1, 2013 to June 31, 2017 (85% of the total samples) and the remaining samples were used for performance testing. The training set only contained 3,613 CIRKP samples, which could cause an unbalancing problem.

Two balancing methods applied in this study were Tomek links (TKL) and Relocating Safe-Level SMOTE (RSLS). The TKL pair is defined as two samples from separate groups that are the closest neighbors (Tomek, 1976), which means there does not exist the third sample that the distances between the third sample and anyone of the TKL pairs are smaller than the distance between the TKL pairs. TKL can balance the training set by removing most class samples from TKL pairs. Besides, removing the whole TKL pairs can also alleviate the invasion problem.

RSLS is a modification of the Safe-Level SMOTE algorithm, which relocates the synthetic sample if the distance between the synthetic sample and a sample from the major class is less than the distance between that synthetic sample and its closest parent sample (Siriseriwan and Sinapiromsaran, 2016). This method considers the surroundings of synthetic samples and can provide a safer oversampling outcome for model training.

This study first removed both samples from all TKL pairs with Euclidean distance on the original feature space to relieve the invasion problem. After that, RSLS was applied to balance the CISKP and CIRKP classes. TKL was implemented with R function “TomeKClassif” in package “UBL,” and RSLS balancing was implemented by calling function “RSLS” in package “smotefamily.”

### Machine Learning Models

Four popular machine learning models were tested in this study including logistic regression (LR), SVM, random forest (RF), and extreme gradient boosting (XGB). The general modeling processing in this study included (1) selecting significant features by multiple statistical methods and (2) constructing models with balancing methods and fivefold cross-validation (except the RF model). The final model included 480 features (472 pseudo-ions, 6 factors for SPC, 1 vector for age, and 1 factor for gender) selected by statistical methods. L1-regularization was applied to the LR model with **l***og*(λ) = −4.77 and finally 102 features were selected in the LR model. The testing performance of the SVM model was achieved by using radial kernel (“rbfdot” kernel in “kernlab” package) with penalty parameter **C** = **1.5**. There were 1,000 trees each with the random sampling size equal to 500 built for the RF model, but the RF model still suffered a serious overfitting problem. The XGB model was set to use “softmax” mode with the max depth equal to 3 and 527 iterations. All the models were implemented with R packages: “glmnet” for LR, “kernlab” for SVM, “randomForest” for RF, and “xgboost” for XGB. Predicting performances were measured by mean area under the receiver operating characteristic curve (AUC) and the accuracy rate of predicting CIRKP, and they are calculated with the assistance of the package “ROCR.” Probability models allow flexible selection of probability cutoff. The optimal probability cutoffs (selected by balancing the specificity and sensitivity of models) of training and testing sets were both shown in this study. In addition, the predicting performance would be analyzed with the optimal cutoff of the training set since the optimal cutoff of the testing set was unobtainable in real antibiotic resistance prediction.

## Results

### Insights for Specimen Information

**FIGURE 2:**
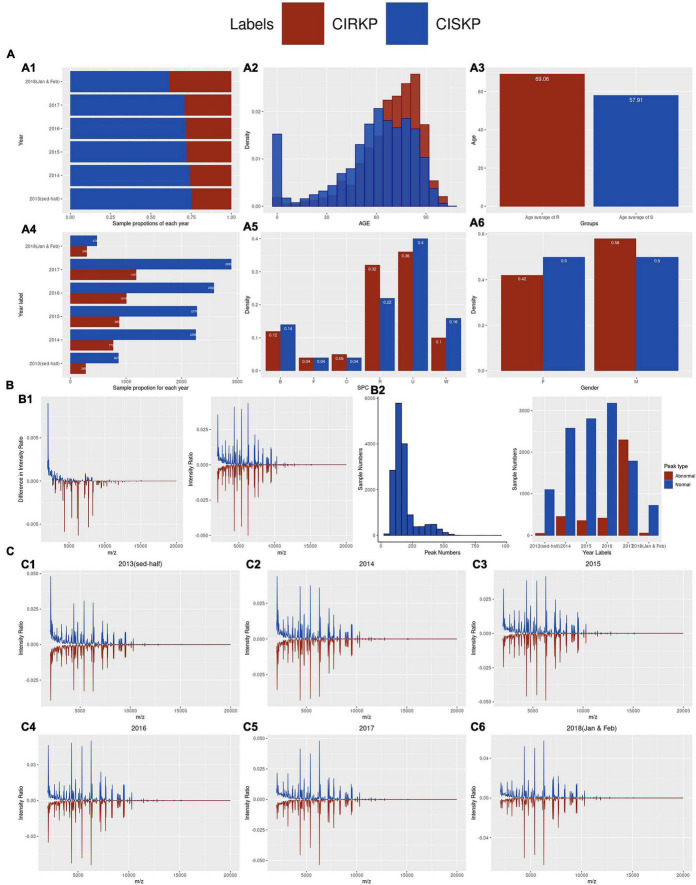
Demographic of statistical information of the data. **(A1,A4)** Proportion of CIRKP samples in each year; **(A2,A3)** age information of samples; **(A5,A6)** number of samples of each SPC and gender in CIRKP and CISKP; **(B1)** overall average spectrum plot of CIRKP and CISKP; **(B2)** distribution of peak numbers. **(C1–C6)** average spectrum plots of CIRKP and CISKP by years.

An increase in the proportion of CIRKP was observed from 25.52% in 2014 to 29.07% in 2017 (Figures 2A1,A4). In total 7,556 (47.87%) and 8,226 (52.12%) samples were obtained from female and male patients. A total of 1,838 (41.51%) female samples and 2,590 (58.49%) male samples comprised the CIRKP group, whereas both female and male samples accounted for around 50% of the CISKP group, suggesting that men were probably more likely to be infected with CIRKP than women (Figure 2A6). Additionally, CIRKP was more likely to be diagnosed in people over the age of 60. The CIRKP group’s average age was 11.15 years older than that of the CISKP group (Figures 2A2,A3). In the case of SPC, more than 60% of samples were collected from respiratory (R) or urinary (U) carriers. Additionally, respiratory (R) and other (O) samples accounted for a greater percentage of samples in the CIRKP group than in the CISKP group (Figure 2A5).

Spectrum data analyzed in this study were collected over the mass-to-charge ratio range from 2,000 to 20,000 m/z. To avoid the problems of magnitudes, the intensity ratio was used for analysis instead of the original intensity in this study. By comparing the average spectrum intensity ratio plot of the CISKP and CIRKP, CRSKP samples were found to have a lower intensity ratio over the lower region of the mass-to-charge ratio (2,000–3,000 m/z) and have a generally higher intensity ratio over the 3,000–7,000 m/z (Figure 2B1). However, no unique spectrum profile or spectrum peak could be observed directly from the average spectrum intensity plot. Moreover, the profiles of the average spectrum intensity ratio of both CISKP and CIRKP were shifting along with the time (Figure 2C). The intensity ratio of peaks at 2,069 m/z decreased from more than 4% to less than 2%. In contrast, peaks at 4,367, 5,382, and 6,291 m/z increased to more than 5% in the first 2 months of 2018. In addition, the numbers of spectrum peaks varied a lot among samples (Figure 2B2). Only 49 peaks were detected from the sample which is the minimum peak number. However, samples with over 900 peaks were also detected. Over 70% of samples contained peaks from 100 to 250. Both the spectrum profile shift and the wide range of peak numbers could indicate that specimens of different sub-strains of *K. pneumoniae* are collected, and their proportions were changing along with time. That was the main reason for the clustering analysis done in the following study.

### Clustering Analysis

Due to the absence of standard MALDI-TOF MS spectrum plots for *K. pneumoniae* sub-strains, it was difficult to determine the true data composition. The clustering approach used in this study aimed at offering information about the composition of the samples under the assumption that bacteria from the same sub-strain have similar spectrum profile.

**FIGURE 3:**
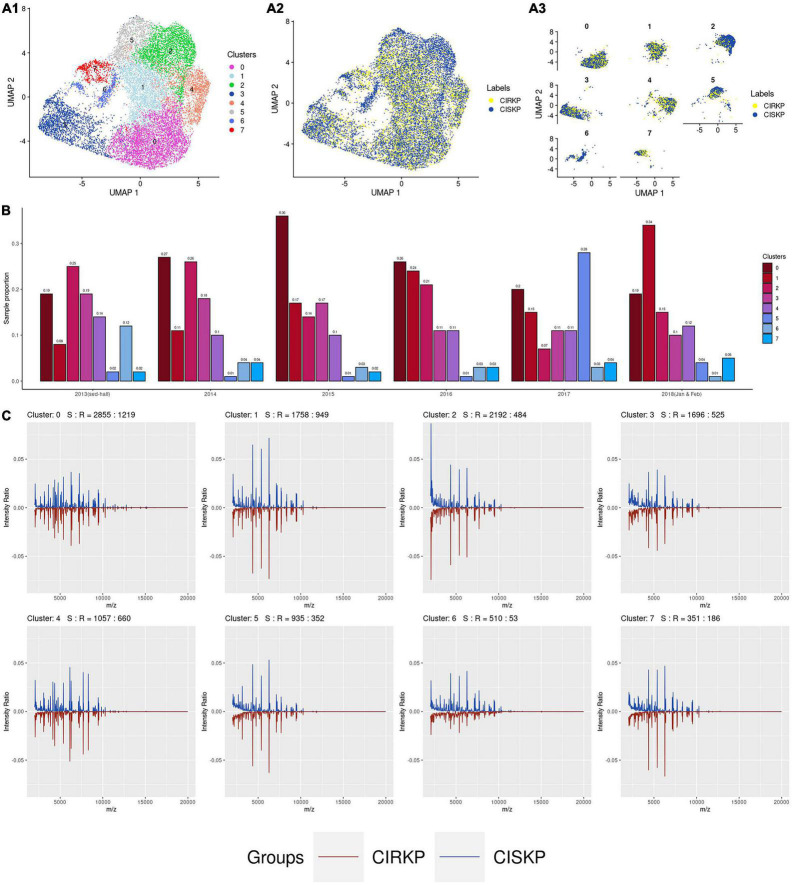
Data visualization of clustering results. **(A1)** Distribution of 8 clusters; **(A2,A3)** distribution of CIRKP and CISKP samples in each cluster; **(B)** proportion of cluster in each year; **(C)** average spectrum plot of each cluster.

By setting the resolution parameter equal to 0.3, eight suspicious sub-strains were detected in this study. The cluster distributions and the distributions of CISKP and CIRKP samples under two-dimensional UMAP reduction are shown in Figures 3A1,A2. Intuitively speaking, clusters 2 and 6 are CISKP-dominant clusters with relatively lower CIRKP proportions compared to other clusters (Figure 3A3). Besides, CIRKP in clusters 2, 5, and 7 seems to be more separable from the CISKP sample than other clusters. But CIRKP and CISKP samples are highly mixed in the other clusters under two-dimensional UMAP reduction. However, no cluster shows a strong relation to the CIRKP or the CISKP group.

The trend of spectrum profile shifts is found from June 2013 to February 2018. It is worth mentioning that cluster 1 has grown from the fifth cluster in 2013 to the biggest cluster in 2018. Combined with the average spectrum plot of cluster 1, we can preliminarily conclude that the increase in the proportion of cluster 1 is the primary cause for the rise in the average intensity ratio of peaks at 4,367, 5,382, and 6,291 m/z (Figures 3B,C). At the same time, the proportion of cluster 0 first significantly increases from 2013 to 2015 and then gradually decreases to the same proportion level of 2013 in 2018. The proportion of cluster 2 keeps decreasing from about 25% of the whole year sample to 15%. As the result of proportion changes of clusters 0, 1, and 2, spectrum peak at 2,069 m/z significantly decreases from 2013 to 2015, then increases a little bit in 2016, and sharply drops to less than 2% in 2018. Moreover, the proportion cluster 5 is found to be abnormally high only in 2017. The majority of cluster 5 are those with abnormally high number of spectrum peaks (peak number 250). Besides, most of the samples with abnormally low peak numbers (peak number 100) are found in cluster 1 (Supplementary Figure 3). For the other clusters, their proportions do not change significantly during the time of specimen collection.

After clustering analysis, it is normal to create classification models for each cluster. However, models trained by samples from all clusters were finally selected rather than models for each cluster. The reasons for this decision include:

1. The clusters in this study only represent suspicious sub-strains of *K. pneumoniae* without any additional support materials, implying that they are unreliable for modeling.
2. Not all cluster-based models outperform the overall model.
3. Most importantly, cluster-based models are unable to handle new samples from unknown clusters.

### Feature Selection

For feature selection, statistical methods were applied on the training set to select associated non-spectrometric features and rank the significance of pseudo-ions.

The Chi-square test was used to determine the statistical significance of non-spectrometric variables using the original gender data as well as the age and SPC data after grouping (Table 1). The results of the tests indicated that CIRKP is associated with all non-spectrometric features. As a result, all dummy variables created from gender and SPC, and the actual age data, would be included in modeling.

**TABLE 1:** Significance of non-spectrometry covariates.

		Sample population			
		
		CISKP	CIRKP	Total number	*P*-value
Total numbers		9,201	3,424	12,625	
Gender (ratio %)					< 2.2×10^−16^
	Male	4,581 (49.7)	2,030 (59.3)	6,611	
	Female	4,620 (50.3)	1,394 (40.7)	6,014	
Age (ratio %)					< 2.2×10^−16^
	Infant	856 (9.3)	41 (1.2)	897	
	Children	50 (0.5)	12 (0.4)	62	
	Teenager	62 (0.7)	14 (0.4)	76	
	Youth	1,201 (13.1)	272 (7.9)	1,473	
	Middle-aged	3,715 (40.4)	1,167 (34.1)	4,882	
	Senium	3,317 (36.0)	1,918 (56.0)	5,235	
Specimen type (ratio %)					< 2.2×10^−16^
	B	1,330 (14.5)	400 (11.7)	1,730	
	F	351 (3.8)	133 (3.9)	484	
	W	1,490 (16.2)	373 (10.9)	1,863	
	R	2,081 (22.6)	1,148 (33.5)	3,229	
	U	3,610 (39.2)	1,276 (37.3)	4,886	
	O	339 (3.7)	94 (2.7)	433	

After the construction of pseudo-ions, significance testing was performed using pseudo-ion vectors. Generally speaking, 18 pseudo-ions and 66 pseudo-ions are found uniquely in CIRKP and CISKP samples. However, only pseudo-ion 434 has been observed 12 times uniquely in CIRKP groups; the other unique pseudo-ions are all observed less than 5 times. Thus, the statistical power of those unique observed pseudo-ions is not guaranteed, and they will not be considered as highly significant features. Wilcoxon rank-sum test and average fold change selection were served for the significance check of mean values. The majority of the 209 pseudo-ions selected by the Wilcoxon rank-sum test concentrate at relatively low-intensity regions with high observation times, but the fold changes of those pseudo-ions are usually insignificant. In contrast, fold change selection selected pseudo-ions of highly different average intensity ratios, usually with lower observation times compared to pseudo-ions selected by the Wilcoxon rank-sum test. *F*-test and KS-test selected 303 and 208 pseudo-ions, respectively, and these pseudo-ions are highly overlapping with the pseudo-ions selected by the Wilcoxon rank-sum test. In conclusion, only pseudo-ions 367 and 407 passed all the selection criteria, and 154 pseudo-ions pass all tests except fold change selection.

The ranks of pseudo-ions were calculated by their average ranks of each selection criterion. The top 15 significant pseudo-ions selected by multiple statistical methods are shown in Table 2. Pseudo-ions that pass one of the four selection criteria were considered significant for modeling and would be used for model construction.

**TABLE 2:** Top 15 significant pseudo-ions selected by statistical methods.

Rank	Pseudo-ion	m/z	Mean difference of peak intensity ratio (10^−4^)	log_2_(fc)	Wilcoxon rank sum test	*F*-test	KS test	Observation times
1	**Pseudo-ion 136**	(4,700, 4,720)	10.22	0.88	1.24×10^−38^	≤0.01	≤0.01	3,200
2	Pseudo-ion 5	(2,080, 2,100)	−50.75	−0.43	1.02×10^−51^	≤0.01	≤0.01	12,528
3	Pseudo-ion 27	(2,520, 2,540)	−13.63	−0.41	1.73×10^−64^	≤0.01	≤0.01	10,704
4	Pseudo-ion 95	(3,880, 3,900)	−3.51	−0.61	2.40×10^−10^	≤0.01	≤0.01	3,358
5	Pseudo-ion 353	(9,040, 9,060)	−1.03	−0.77	5.60×10^−05^	≤0.01	≤0.01	**478**
6	Pseudo-ion 1	(2,000, 2,020)	−13.52	−0.40	1.72×10^−41^	≤0.01	≤0.01	10,727
7	Pseudo-ion 32	(2,620, 2,640)	−13.09	−0.40	1.01×10^−34^	≤0.01	≤0.01	10,453
8	Pseudo-ion 293	(7,840, 7,860)	−0.26	−0.67	3.20×10^−07^	≤0.01	≤0.01	**406**
9	Pseudo-ion 284	(7,660, 7,680)	−4.76	−0.72	1.87×10^−03^	≤0.01	≤0.01	2,873
10	Pseudo-ion 11	(2,200, 2,220)	−13.45	−0.37	6.23×10^−42^	≤0.01	≤0.01	10,405
11	Pseudo-ion 2	(2,020, 2,040)	−20.51	−0.38	9.96×10^−34^	≤0.01	≤0.01	11,433
12	Pseudo-ion 4	(2,060, 2,080)	−97.53	−0.39	2.46×10^−23^	≤0.01	≤0.01	12,318
13	**Pseudo-ion 367**	(9,320, 9,340)	0.01	1.11	3.86×10^−04^	≤0.01	0.01	**285**
14	Pseudo-ion 50	(2,980, 3,000)	−12.41	−0.35	6.23×10^−33^	≤0.01	0.01	10,574
15	Pseudo-ion 163	(3,240, 3,260)	−3.71	−0.84	2.46×10^−09^	≤0.01	0.01	**768**

*Mean difference is calculated by CIRKP-CISKP;fc represents the fold change value; fold change is calculated by CIRKP/CSIKP; total number of training samples: 13,414. Bold type values means the statistical quality of these pseudo-ions are relatively lower than other pseudo-ions since less samples are observed.*

### Classification Result

**FIGURE 4:**
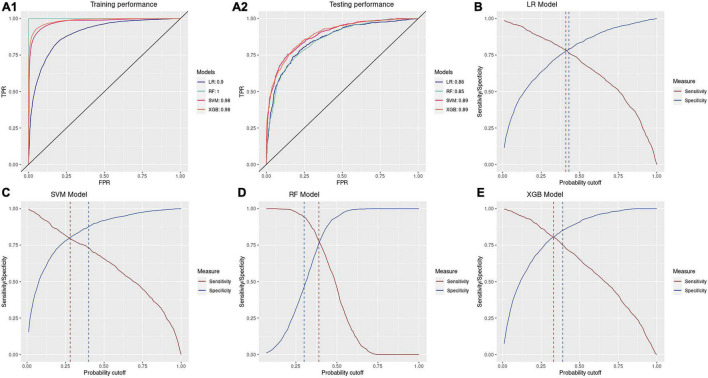
Performance of models. **(A1,A2)** Training and testing ROC plots of four models; **(B–E)** optimal probability cutoff of training and testing set for four models.

All four models showed high accuracy in detecting antibiotic resistance of patients. Both SVM and XGB have high AUC values of 0.89 on the testing set with specificities and sensitivities of 0.80 under the optimal probability cutoff of the testing set (Figure 4). Under the optimal cutoff of the training set, XGB and SVM could achieve accuracies of 0.82 [95% CI: (0.80, 0.83)] and 0.83 [95% CI: (0.81, 0.84)] of predicting CIRKP and CISKP samples in the testing set. The testing AUC of LR and RF is around 0.86 and 0.85, respectively. However, due to the severe overfitting problem of RF, the predicting performance under the optimal probability cutoff of the training set is highly unbalanced, which achieves an extremely high sensitivity of over 0.94 but low overall accuracy of 0.63 and unacceptable specificity of 0.47. Compared to RF, LR performs more stably. The gap between optimal cutoff of training and testing sets for LR is 0.02, which means LR is the only one of four models that could achieve both predicting sensitivity and specificity around 0.76 under the optimal cutoff of the training set time. However, since SVM and XGB could also achieve sensitivities of 0.73 and 0.75 and specificity of 0.88 and 0.85 simultaneously, they are considered slightly better model choices than LR.

Four models perform well on identifying CIRKP. We were interested in the differences between significant features selected by models and statistical methods (Table 3). The absolute value of coefficients directly ranked the feature significance of LR. The mean of decreasing in the Gini index was used to rank features that construct RF. The feature ranks for SVM and XGB models were created by constructing new models without target features and calculating the decrease in testing AUC. For the results, only pseudo-ions 136 and 367 are considered the top 15 significant pseudo-ions by both models and statistical methods. Pseudo-ion 171 is also considered as one of the top 15 features by three models except for LR. Meanwhile, both LR and statistical method ranked pseudo-ion 171 at about 70th. Considering the 49 amino acids protein AcrZ subunit of AcrAB efflux pump whose expression change is proved to be one of the mechanisms of ciprofloxacin resistance, pseudo-ion 171 may be a representation of the expression level of AcrZ (Pakzad et al., 2013; UniProt Consortium et al., 2021). The statistical rank for features is very different from their real contribution in models. Statistical methods and each model select less than 10 identical top 100 features, respectively. However, feature contributions in RF and XGB models are highly consistent. Sixty-two identical features are found among their top 100 features. This observation could indicate that a better selection method or feature ranking system may help improve the classification outcome.

**TABLE 3:** Top 15 significant features selected by models.

	Features (observed sample number)														

	Rank 1	Rank 2	Rank 3	Rank 4	Rank 5	Rank 6	Rank 7	Rank 8	Rank 9	Rank 10	Rank 11	Rank 12	Rank 13	Rank 14	Rank 15
LR	AGE (13,414)	GEN (13,414)	U (5,218)	PI138 (9,756)	R (3,408)	PI172 (6,666)	PI307 (4,831)	PI228 (1,694)	PI158 (13,137)	PI495 (1,358)	PI103 (3,940)	PI203 (551)	PI290 (2,367)	PI287 (4,403)	PI92 (8,767)
SVM	PI154 (3,324)	AGE (13,414)	PI36 (3,200)	PI226 (5,434)	PI494 (2,858)	PI171 (9,543)	PI127 (1,739)	PI306 (5,431)	PI273 (606)	PI103 (3,940)	PI1495 (1,358)	PI230 (7,252)	PI367 (285)	PI198 (4,580)	PI52 (10,181)
RF	AGE (13,414)	PI171 (9,543)	R (3,408)	PI154 (3,324)	GEN (13,414)	PI136 (3,200)	PI208 (5,405)	PI91 (8,789)	PI288 (12,122)	PI165 (9,641)	PI316 (9,601)	PI226 (5,434)	PI108 (9,883)	PI286 (12,699)	PI306 (5,431)
XGB	R (3,945)	AGE (13,414)	GEN (13,414)	PI171 (9,543)	PI136 (3,200)	PI54 (3,324)	PI208 (5,405)	PI91 (8,789)	PI226 (5,434)	PI306 (5,431)	PI31 (9,619)	PI165 (9,641)	PI266 (5,375)	PI316 (9,601)	PI288 (12,112)

*PI, pseudo-ion; GEN, gender; R, SPC-R; U, SPC-U.*

As for the non-spectrometric features, age is the most significant feature selected by models, constantly ranked as the top 2 significant features. Besides, gender is another highly significant feature ranked in the top 3 by LR, RF, and XGB models. These results show that patients’ basic information may also contribute a lot to the real antibiotic resistance identification problems. Among all SPC types, only respiratory SPC (SPC-R) is considered one of the top 5 features of all models except SVM. Urinary SPC (SPC-U) is the third critical feature in LR, and it also ranked in the top 100 in the other models. Excluding SPC-U and SPC-R, other SPC types are not incredibly important for models. Besides, categorical variables are less popular for SVM compared to the other three models.

## Discussion

### Analysis of In-Cluster Performance

Instead of constructing cluster-based models, four overall models constructed in this study utilized data from all clusters as training and testing samples. The overall performance of models showed high accuracy, and we wondered whether these models were competent for predicting antibiotic resistance for multiple suspicious sub-strains of *K. pneumoniae* (Table 4).

**TABLE 4:** In cluster performance of the general model.

	Training (AUC %)				Testing (AUC %)			
		
Clusters	LR	SVM	RF	XGB	LR	SVM	RF	XGB
**Overall**	**89.69**	**97.61**	**100.00**	**98.50**	**85.58**	**88.86**	**85.44**	**89.08**
0	90.61	98.00	100.00	98.84	82.89	85.70	84.27	85.95
1	90.56	97.58	100.00	98.82	84.82	87.70	86.34	89.80
2	89.25	97.55	100.00	98.24	92.16	93.72	90.74	91.03
3	89.14	97.47	100.00	98.41	**80.05**	84.12	**79.41**	83.15
4	89.01	97.45	100.00	98.28	80.84	87.31	81.43	86.70
5	88.26	96.73	100.00	98.55	88.95	91.44	87.94	91.31
6	87.10	96.70	100.00	97.22	**50.00**	92.59	**74.07**	**57.41**
7	91.15	97.63	100.00	98.73	85.30	88.67	87.98	93.81

*Bold type AUC value of cluster 0–7 shows poor performances of machine learning models on those clusters.*

Four models can manage the in-cluster predicting task with acceptable performance most of the time. All four models exhibit high testing performance on predicting CIRKP in clusters 2, 5, and 7. This observation is consistent with the clustering analysis that these three clusters are more separable than other clusters. Furthermore, the outstanding performance on cluster 5 indicates that samples with abnormally high peak numbers are separable, and it is unnecessary to remove those samples from analysis. Compared to the other three models, SVM shows high stability in handling classification tasks on different clusters with the lowest AUC value of 0.84. Nevertheless, poor performances could also be found in predicting CIRKP on clusters 3 and 6. The predicting AUC value of LR and XGB are only 0.50 and 0.57 on cluster 6, but the training AUC achieves 0.87 and 0.97, respectively, which seems that both LR and XGB cannot perform the classification task better than a model performs random selection. The predicting AUC of RF on cluster 6 also significantly drops 0.74. However, the low AUC values on cluster 6 are not due to the failure of models. By looking into the confusion matrixes under the optimal probability cutoff of the training set for each cluster (Table 5), only 1 CRIKP sample is found in the testing set of cluster 6. That means the AUC value of cluster 6 is unreliable. All models could achieve overall accuracy of over 0.92 except RF. As for model performances on cluster 3, LR, SVM, and XGB all show high specificity but low sensitivity. Even for RF, which achieves an overall sensitivity of 0.94, it cannot excellently classify CIRKP samples in cluster 3. The relatively inferior performance on cluster 3 provides evidence for the potential influence of sub-strains on the model.

**TABLE 5:** Confusion matrix of each model with optimal probability cutoff of training set.

Real predicted		Cluster 0		Cluster 1		Cluster 2		Cluster 3	
					
		CIRKP	CISKP	CIRKP	CISKP	CIRKP	CISKP	CIRKP	CISKP
LR	CIRKP	127	62	166	97	15	6	38	35
	CISKP	41	206	29	167	5	91	24	140
SVM	CIRKP	117	36	156	49	16	2	36	18
	CISKP	51	232	39	215	4	95	26	157
RF	CIRKP	**160**	**160**	**192**	**209**	**17**	**19**	**49**	**69**
	CISKP	**8**	**108**	**3**	**55**	**3**	**78**	**13**	**106**
XGB	CIRKP	105	37	161	57	14	5	35	22
	CISKP	63	231	34	207	6	92	27	153

		**Cluster 4**		**Cluster 5**		**Cluster 6**		**Cluster 7**	
					
		**CIRKP**	**CISKP**	**CIRKP**	**CISKP**	**CIRKP**	**CISKP**	**CIRKP**	**CISKP**

LR	CIRKP	91	46	159	57	**0**	**3**	28	20
	CISKP	22	103	61	413	**1**	**51**	8	55
SVM	CIRKP	85	27	156	37	**0**	**2**	30	21
	CISKP	28	122	64	433	**1**	**52**	6	54
RF	CIRKP	**111**	**129**	**202**	**187**	**0**	**11**	**36**	**49**
	CISKP	**2**	**20**	**18**	**283**	**1**	**43**	**0**	**26**
XGB	CIRKP	93	36	169	50	**0**	**2**	34	20
	CISKP	20	113	51	420	**1**	**52**	2	55

		**LR**		**SVM**		**RF**		**XGB**	
					
**REF**		**CIRKP**	**CISKP**	**CIRKP**	**CISKP**	**CIRKP**	**CISKP**	**CIRKP**	**CISKP**

	CIRKP	624	326	596	192	**767**	**825**	611	229
	CISKP	191	1,226	219	1,360	**48**	**727**	204	1,323

*RF model is severely overfitted to CIRKP group. The in-cluster performance of cluster 6 is acceptable but low AUC value is caused by insufficient positive test samples.*

In conclusion, four overall models constructed in this study can manage the classification task of CIRKP for most suspicious sub-strains, but the potential risk of sub-strain effect still exists. Determining the antibiotic resistance results by the optimal cutoff of each suspicious sub-strain instead of using the overall optimal cutoff of the training set may alleviate effect. However, a rigorous sub-strains identification process and solution to managing newly observed sub-strains should be set up.

### Time Influence

**FIGURE 5:**
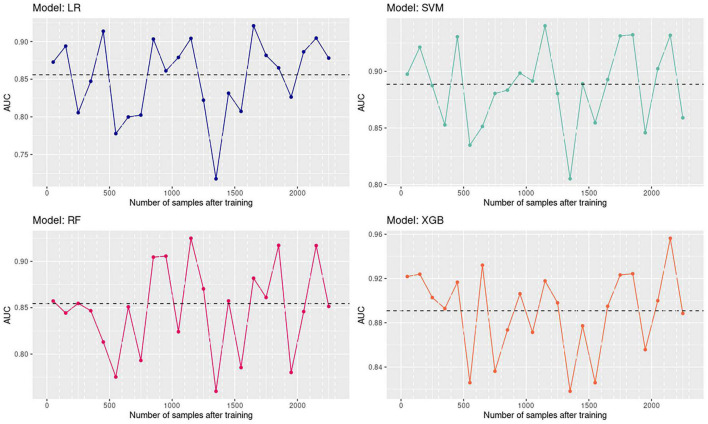
Time influence on the performance of four models.

Time influence on the predicting performance was considered by analyzing the fluctuation of the AUC value as the testing time period got far away from the last training samples. The first 2,300 samples in the testing sets were grouped into 23 groups by time, with 100 samples in each group. After that, the predicting AUC of each testing group is calculated and plotted in Figure 5. The expected decreasing trend in model performance along with time is not observed. Moreover, the same trend of the AUC changes is found among four models, which means the predicting performance of models is highly related to the separability of current samples. That emphasizes that even machine learning models with high classification accuracy in the past may fail in the current antibiotic resistance identification task. Reasons for the time effect could also include that the overall model cannot perform well on all the potential sub-strains, but the sample composition varies along with time.

Four models constructed in this study show high accuracy in identifying CIRKP. Especially SVM and XGB, which perform stably on all clusters and exhibit AUC greater than 0.8 for all the testing time periods. However, the stability against time effect could also be an essential criterion for clinically used antibiotic resistance predicting models.

## Importance and Conclusion

The rising prevalence of CIRKP has increased the risk of incorrect selection of initial antibiotic treatment. The purpose of this study is to develop machine learning-based models for identifying antibiotic resistance of CIRKP using data from a longitudinal study done from June 2013 to February 2018. The use of large-scale data ensured the statistical quality of the selected biomarkers. Significant differences between the CISKP and CIRKP of a few pseudo-ions in the high mass-to-charge range were also detected, but their observation times were insufficient to draw a firm conclusion. Both statistical approaches and modeling algorithms recommended expanding the training set to conduct reliable statistical results for all the pseudo-ions. That indicated the need for a systematic and comprehensive MALTOF-MS database. Additionally, models were trained and evaluated in this work utilizing spectrum data from June 2013 to June 2017 and extra non-spectrometric information. Future samples were used to replicate the real-world antibiotic resistance prediction issue. Clustering analysis and the effect of time on model performance were implemented to provide potential information of suspicious sub-strains and demonstrate overall models’ problems. While the model’s performance does not meet the clinical standard, the findings of this investigation confirm the potential usefulness of the machine learning-based approach for antibiotic identification.

Limitations of this study include two main points, which are (1) the models may not be able to generalize to other bacterial species and antibiotics, and (2) the absence of biological validations. Protein analysis may be used to confirm the biological importance of selected features, and thorough real strain analysis, rather than the suspect sub-strain analysis used in this work, would give more helpful information. However, it is challenging to verify accurate sub-strains information in this study due to the data limitations. Additionally, other advanced machine learning techniques, such as deep learning and integrated prediction of multiple models, may enhance prediction accuracy.

## Data Availability Statement

The original contributions presented in the study are included in the article/Supplementary Material, further inquiries can be directed to the corresponding author/s.

## Author Contributions

T-YL and ZW contributed to the study concept and design. H-YW and J-JL assisted in the data acquisition. CW and ZW carried out the data analysis. CW was response for drafted the manuscript. C-RC, J-TH, and J-JL helped conceive the study. All authors contributed to the data interpretation and revised the manuscript.

## Conflict of Interest

The authors declare that the research was conducted in the absence of any commercial or financial relationships that could be construed as a potential conflict of interest.

## Publisher’s Note

All claims expressed in this article are solely those of the authors and do not necessarily represent those of their affiliated organizations, or those of the publisher, the editors and the reviewers. Any product that may be evaluated in this article, or claim that may be made by its manufacturer, is not guaranteed or endorsed by the publisher.
