# Supplementary material for: Large-Scale Samples Based Rapid Detection of Ciprofloxacin Resistance in Klebsiella pneumoniae Using Machine Learning Methods
Source: Front Microbiol. 2022 Mar 8;13:827451. doi: 10.3389/fmicb.2022.827451 (PMC8959214; doi:10.3389/fmicb.2022.827451)
Supplement: Supplementary file 2 [file Table_1.pdf]

Supplementary Table 1	
**SPC**	**Details**
R	SP, BW, BAL, BA
U	U, UU, PCN, FU, SPA
F	DIA, AS, TIP, PL, BF, SY, CPD, LV
W	PUS, WD, AB, TS, OTH, SSS, AM, BO, BM, CX, VA
B	B, CVP, DL
O	Others

Detailed types of specimen carrier for each abbreviation

Supplementary Table 2	
**Age group**	**Age(x)**
Infant	$0 \leq x < 7$
Children	$7 \leq x < 13$
Teenager	$13 \leq x < 18$
Youth	$18 \leq x < 46$
Middle-age	$46 \leq x < 70$
Senium	$x \geq 70$

Detailed age range for each age label

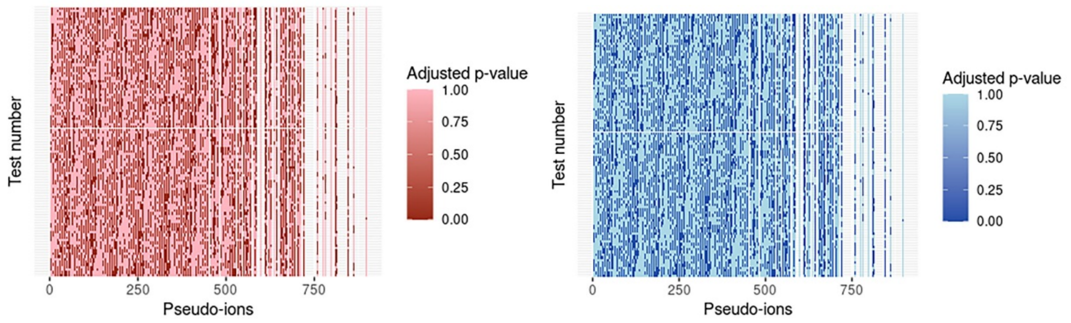

Supplementary plots 1 & 2: Normality test (Shapiro-Wilk Test) results of CIRKP (red) and CISKKP (blue).

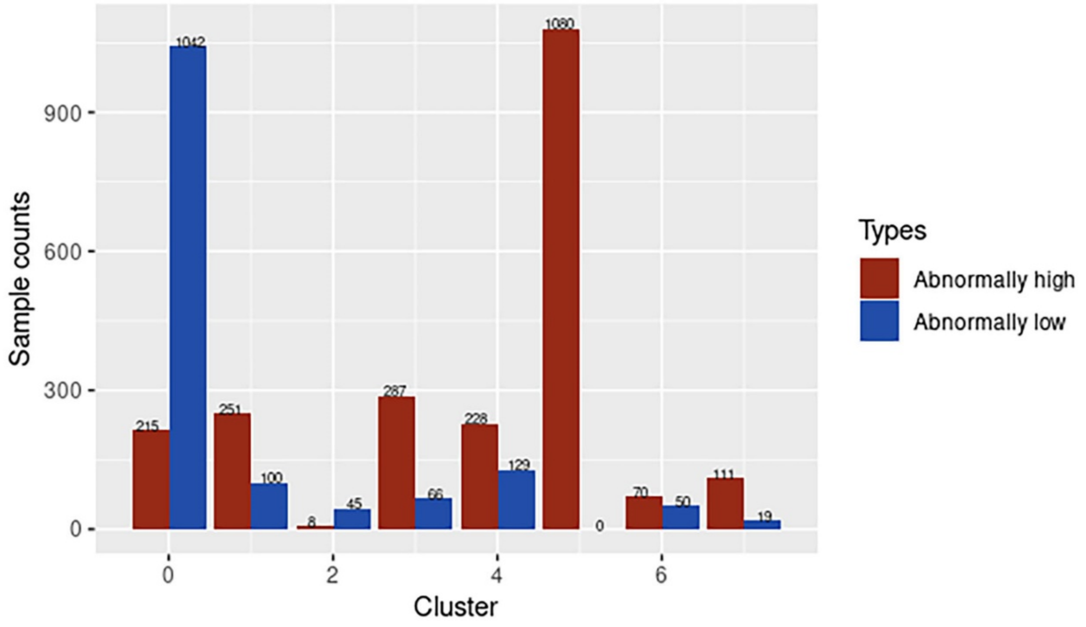

Supplementary plot 3: The number of samples with abnormally high and abnormally low number of spectrum peaks in each cluster.

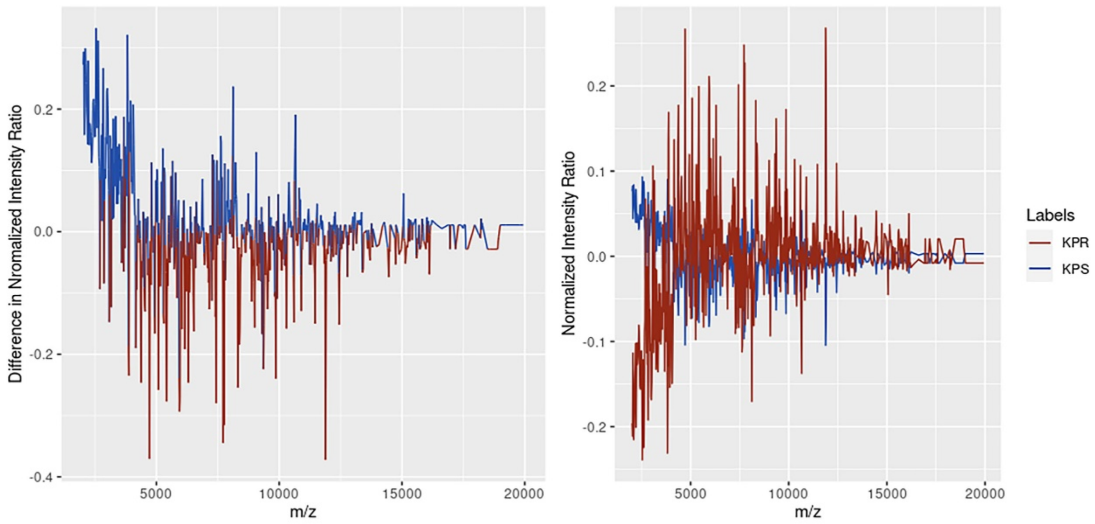

Supplementary plot 4: Average spectrum plots of CRIKP and CISKP after normalization.
