# Supplementary figures and images for: Large-Scale Samples Based Rapid Detection of Ciprofloxacin Resistance in Klebsiella pneumoniae Using Machine Learning Methods
Source: Front Microbiol. 2022 Mar 8;13:827451. doi: 10.3389/fmicb.2022.827451 (PMC8959214; doi:10.3389/fmicb.2022.827451)

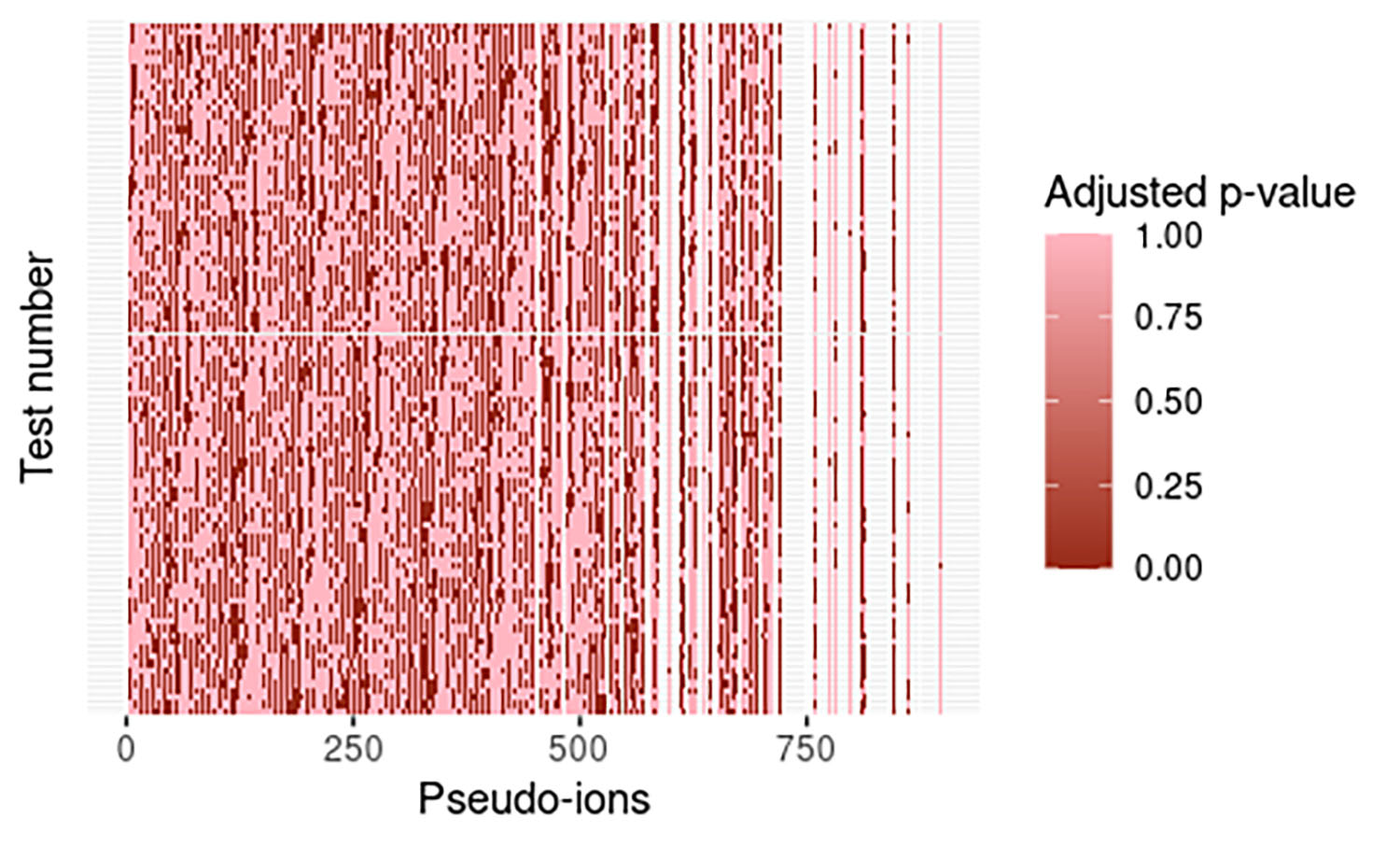

Supplement: Supplementary file 3 [file Image_1.jpg]

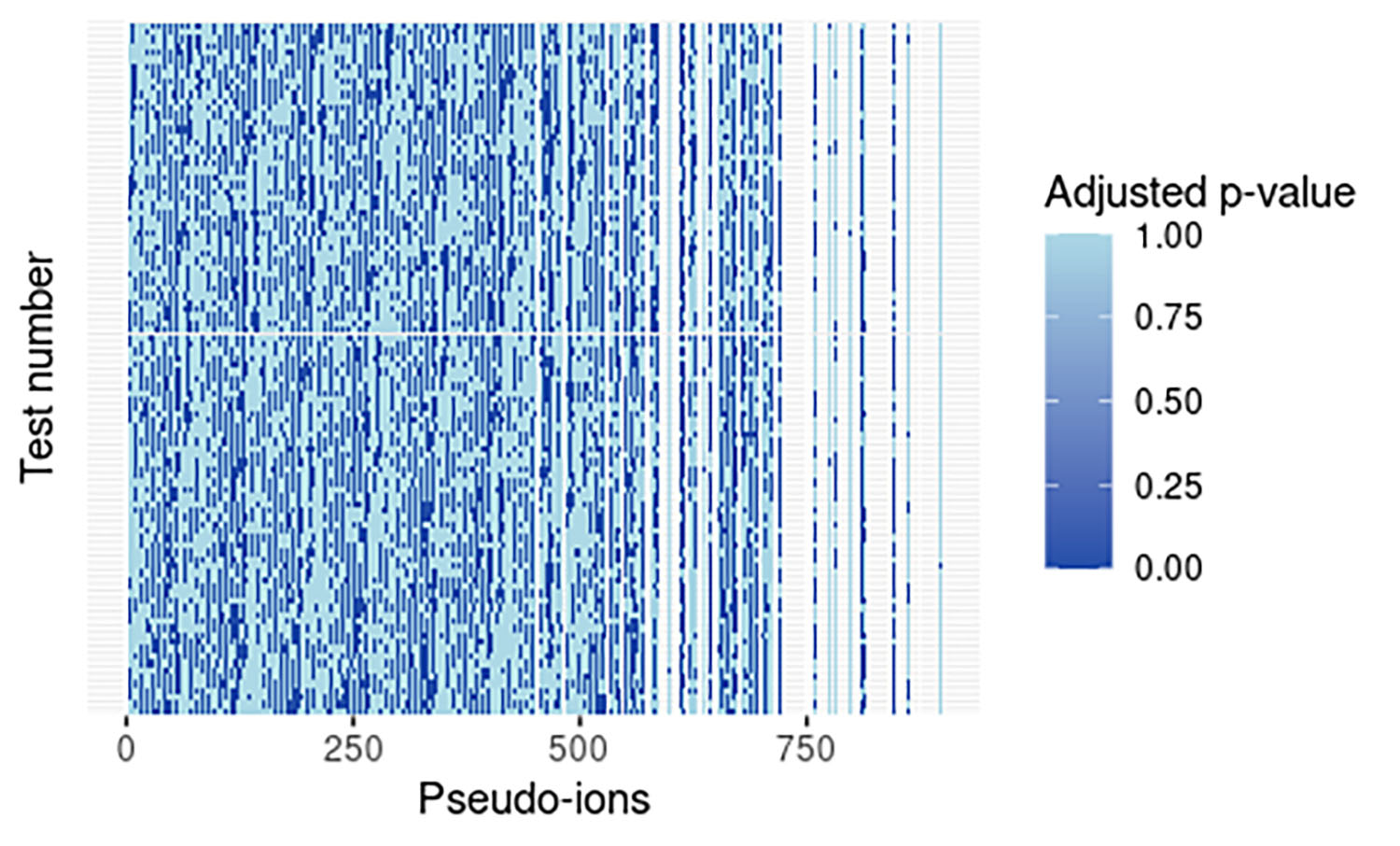

Supplement: Supplementary file 4 [file Image_2.jpg]

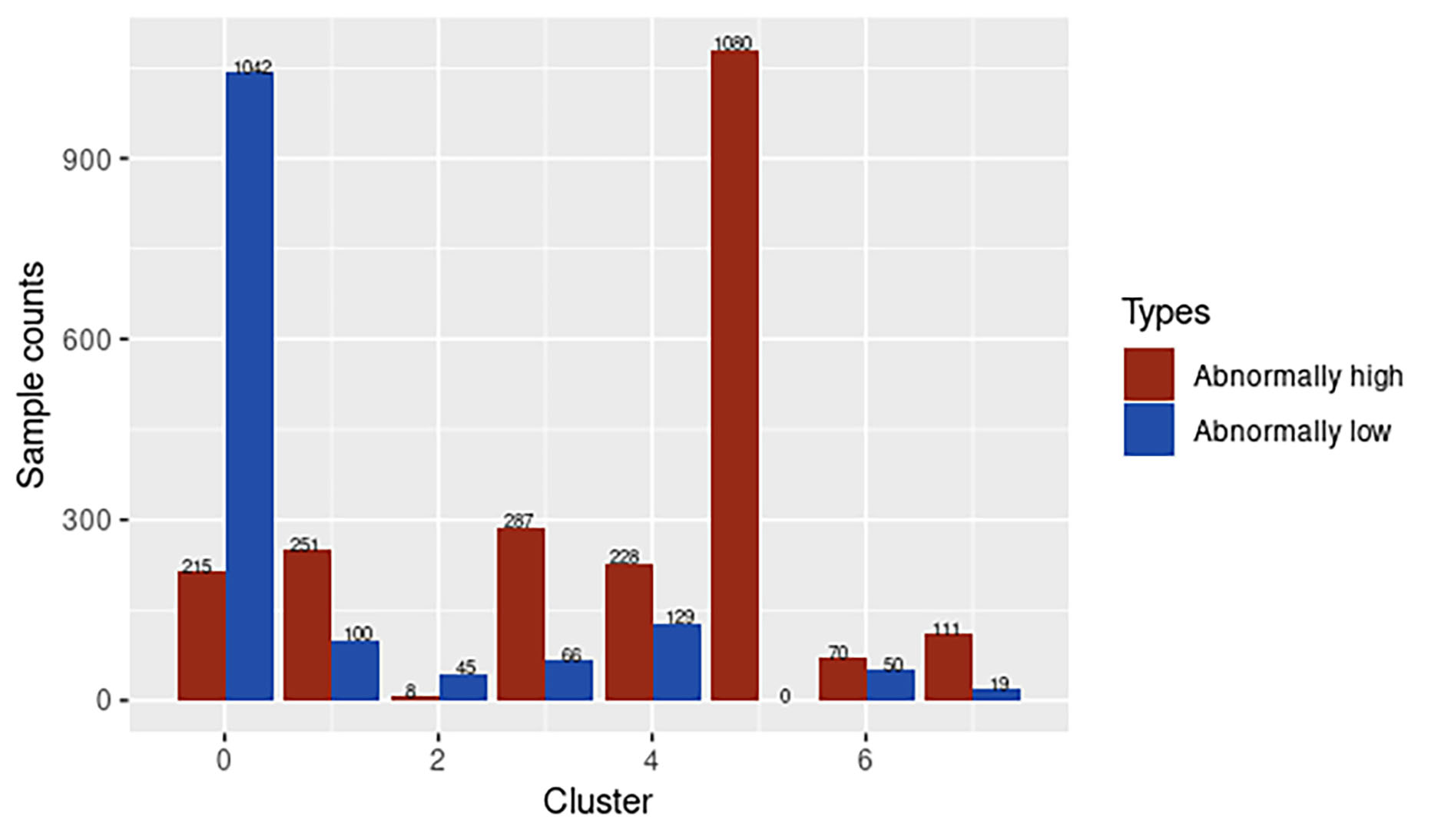

Supplement: Supplementary file 5 [file Image_3.jpg]

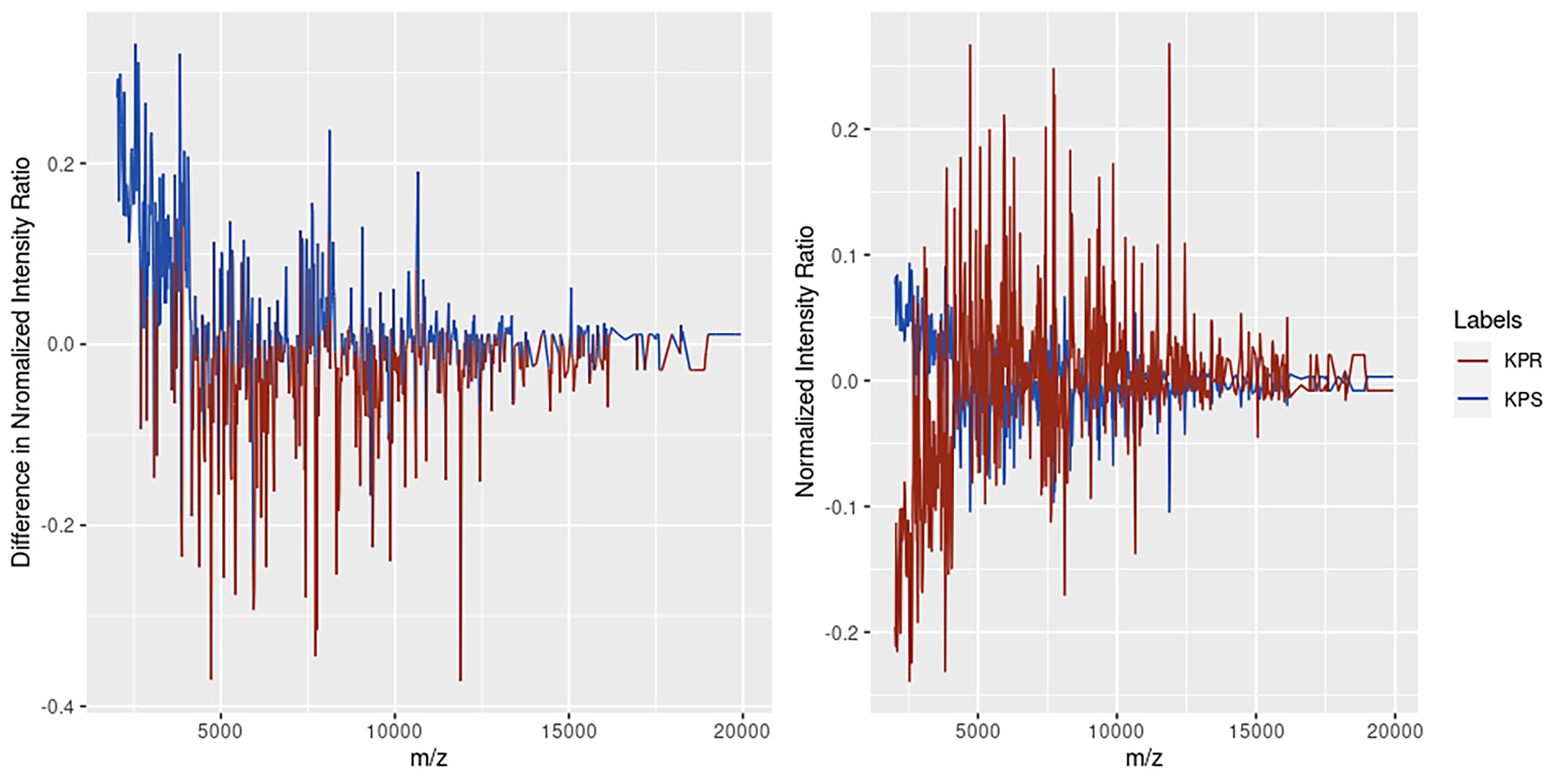

Supplement: Supplementary file 6 [file Image_4.jpg]
